# Structure-based mechanism and inhibition of cholesteryl ester transfer protein

**DOI:** 10.1007/s11883-023-01087-1

**Published:** 2023-03-07

**Authors:** Han Xue, Meng Zhang, Jianfang Liu, Jianjun Wang, Gang Ren

**Affiliations:** 1 The Molecular Foundry, Lawrence Berkeley National Laboratory, Berkeley, CA USA; 2 Beijing National Laboratory for Molecular Science, Institute of Chemistry, Chinese Academy of Sciences, Beijing, China

**Keywords:** Cholesteryl ester transfer protein (CETP), CETP structure, CETP dynamics, Lipoprotein, Low-density lipoprotein (LDL), High-density lipoprotein (HDL), CETP inhibitor, Electron microscopy (EM)

## Abstract

**Purpose of Review:**

Cholesteryl ester transfer proteins (CETP) regulate plasma cholesterol levels by transferring cholesteryl esters (CEs) among lipoproteins. Lipoprotein cholesterol levels correlate with the risk factors for atherosclerotic cardiovascular disease (ASCVD). This article reviews recent research on CETP structure, lipid transfer mechanism, and its inhibition.

**Recent Findings:**

Genetic deficiency in CETP is associated with a low plasma level of low-density lipoprotein cholesterol (LDL-C) and a profoundly elevated plasma level of high-density lipoprotein cholesterol (HDL-C), which correlates with a lower risk of atherosclerotic cardiovascular disease (ASCVD). However, a very high concentration of HDL-C also correlates with increased ASCVD mortality. Considering that the elevated CETP activity is a major determinant of the atherogenic dyslipidemia, i.e., pro-atherogenic reductions in HDL and LDL particle size, inhibition of CETP emerged as a promising pharmacological target during the past two decades. CETP inhibitors, including torcetrapib, dalcetrapib, evacetrapib, anacetrapib and obicetrapib, were designed and evaluated in phase III clinical trials for the treatment of ASCVD or dyslipidemia. Although these inhibitors increase in plasma HDL-C levels and/or reduce LDL-C levels, the poor efficacy against ASCVD ended interest in CETP as an anti-ASCVD target. Nevertheless, interest in CETP and the molecular mechanism by which it inhibits CE transfer among lipoproteins persisted. Insights into the structural-based CETP-lipoprotein interactions can unravel CETP inhibition machinery, which can hopefully guide the design of more effective CETP inhibitors that combat ASCVD.

**Summary:**

Individual-molecule 3D structures of CETP bound to lipoproteins provide a model for understanding the mechanism by which CETP mediates lipid transfer and which in turn, guide the rational design of new anti-ASCVD therapeutics.

## Introduction

Risk factors for atherosclerotic cardiovascular disease (ASCVD) correlate with plasma lipoproteins-cholesterol concentrations [1–4], which can be modulated by cholesteryl ester transfer protein (CETP) activity and its inhibition [5]. CETP is a 476-amio acid residue hydrophobic glycoprotein with a molecular weight of ~53 kDa [6]. As a member of the lipid-transfer protein family, CETP plays a key role in reverse cholesterol transport (RCT) by mediating the transfer of cholesteryl esters (CEs) and triglycerides (TGs) between high density lipoproteins (HDLs) and apolipoprotein B-100 (apoB-100) containing lipoproteins in plasma [7]. Genomic studies have shown that some CETP single-nucleotide polymorphism (SNPs) modify the plasma lipid profiles by altering responses to diet [8]. SNPs with loss of CETP activity are respectively associated with elevated and reduced HDL-cholesteryl (HDL-C) and LDL-cholesterol (LDL-C) concentrations [9]. Moreover, CETP also raises brain cholesterol content [10••]. Thus, CETP inhibition emerged as a strategy for reducing ASCVD events via its effects on plasma HDL-C and LDL-C concentrations [11, 12, 13••]. Unexpectedly, five large clinical trials of CETP inhibitors, i.e., torcetrapib [14], dalcetrapib [15], evacetrapib [16], anacetrapib [17], and obicetrapib [18] were terminated [13••, 19, 20••], for lack of efficacy, no reduction of ASCVD events, suggesting that CETP function and its inhibitory mechanism were inadequately understood [21–23], particularly at a molecular level [24•]. Since the discovery of CETP in the 1980s [25–28], three mechanistic models have been put forward for the CETP-mediated CE transport: (i) the shuttle model: CETP binds to HDL, extracts the CEs from HDL core after which the eCETP-CE complex desorbs from the HDL surface and diffuses to LDL/VLDL and exchanges its complement of CEs for glycerol lipids–phospholipids and especially TGs from LDL/VLDL core before returning to HDL to release the glycerolipids before initiating the next cycle of CE transfer [29]; in this model, only binary complexes of CETP-lipoprotein are formed. (ii) the tunnel model: a ternary complex in which CETP bridges HDL and LDL/VLDL and exchanges HDL-CE for mostly VLDL-TG, via a hydrophobic tunnel within CETP [30]; (iii) the dimer tunnel model: a modified tunnel model, in which the hydrophobic tunnel is formed by a CETP dimer instead of monomer [31]. Although these models account for the basic lipid transport process [32], the detailed mechanism is unknown due to the dynamic properties and structural flexibilities of lipoprotein and CETP-lipoprotein complexes [33, 34••, 35–37].

Recently, individual particle electron tomography (IPET) was developed and used to study the individual-molecule 3D structure [34••] via a “snap-shot” 3D density map of an individual protein particle or protein complex (non-averaging) [34••, 35, 38–49]. The visualization of CETP-lipoprotein complex sheds light on the CE transfer mechanism at a molecular level [26, 42]. Here, we reviewed the structure-based mechanism of CETP function in CE transport, focusing on the CETP-lipoprotein interaction, to understand CE transfer and its inhibition for a better inhibitor design in preventing and reversing ASCVD.

## The Physiological Role of CETP in Plasma Lipid Transport

### Lipid Transfer Protein Family

CETP belongs to the lipid transfer protein (LTP) family [50] that includes other members—phospholipid transfer protein (PLTP) [51], lipopolysaccharide-binding protein (LBP) [52], bactericidal/permeability increasing protein (BPI) [53, 54], ceramide-transfer protein (CERT) [55], sphingolipid-transfer proteins (CERT and FAPP2), phosphatidylcholine-transfer protein (PCTP) [56], phosphatidylinositol-transfer protein (PITP) [57], retinoid binding proteins (RBPs) [58], and α-tocopherol transfer protein [59]. These family members share sequence and structural homology [60], and although each member mediates a distinct physiological process, they all bind lipopolysaccharides and phospholipids [61]. Due to these activities in promoting the exchange of neutral lipids and phospholipids between the plasma lipoproteins [62], CETP and PLTP have raised some attention as a potential drug target to treat ASCVD.

### Physiological Function

In humans, CETP is mainly expressed in the liver, small intestine, adipose tissue, and spleen [63]. The expression of the CETP gene is stimulated by dietary cholesterol and endogenous hypercholesterolemia [64]. The low concentration of human plasma CETP, ~2 μg/mL [31], mediates CE and TG transfer between plasma HDL particles and apolipoprotein B (apoB)-containing lipoprotein particles (e.g., VLDL and LDL) during lipoprotein metabolism [9, 31, 36, 65]. Plasma CE transfer activity depends on both CETP concentration and CETP efficiency [66, 67]. A higher level of CETP correlates with a lower level of HDL formation [9, 68]. Aside from its established role in transferring CEs, CETP also modulates plasma lipid the RCT pathway [69]. In ASCVD, CETP activity increases the LDL-C and apoB concentrations, most likely a consequence of downregulated hepatic LDL receptors [31]. The smaller, denser LDLs may be more atherogenic than normal, “fluffy” LDL due to its higher affinity for artery-wall proteoglycans and greater susceptibility to oxidation [9]. In contrast with these pro-atherogenic effects, CETP remodels HDL particles in a way that releases lipid-poor apolipoprotein A-1 (APOA1) [70]. One direct benefit of CETP inhibition is a reduced cholesterol uptake and an increased cholesterol efflux by cells within atherosclerotic plaques [19].

Genetic CETP deficiency markedly increases HDL particle size and number [71, 72], and half-normal distributions of CETP have a similar but less profound effect that is associated with increased plasma APOA1 concentrations [71, 72]. Studies of patients heterozygotic for a CETP mutation correlated with fewer ASCVD events and elevated HDL-C concentrations [73]. Although these studies suggest that CETP inhibition is atheroprotective, subsequent studies on heterozygotes in the families with CETP deficiency revealed no evidence of premature atherosclerosis [71, 72], despite its regulation of HDL subclass 2 [71, 72]. Moreover, the genetic studies showed that CETP deficiency is an independent ASCVD risk factor [74], a finding that was inconsistent with strategies to reduce ASCVD via CETP inhibition. Nevertheless, the recent combination analysis of three CETP gene SNPs among 27,196 CHD showed CETP genotypes are associated with moderate inhibition of CETP activity and modestly higher HDL-C levels, which is weakly, inversely associated with ASCVD risk [75]. Unlike LDL, which is mechanistically linked to premature ASCVD, HDL is only an ASCVD modifying factor [76].

### PLTP Biochemical Function

PLTP mediates the phospholipid transfer between HDL and LDL (VLDL) [62, 77, 78], and has a unique role in HDL remodeling. Better understanding the PLTP mechanism may guide our understanding of the CETP mechanism [79] and its role in ASCVD [80, 81]. According to EM and IPET techniques, PLTP, like CETP, has a banana-shaped structure [41], that successively penetrates HDL and LDL surfaces, thereby forming a ternary HDL-PLTP-LDL complex that mediates phospholipid transfer in a way that seems more complex than that for CETP [41]. The specific activity of PLTP is higher than that of CETP in terms of HDL fusion into a larger size, which occurs by an unknown mechanism [41]. The similar structure with different activities implies that a few amino acid residues determine specificity. Identification of the mechanism by which PLTP mediates HDL fusion could help refine CETP mechanism. Comparisons of PLTP and CETP sequences and activities could reveal key structures within each protein determine specificity.

## Molecular Structures of CETP

As described in detail below, the structures of CETP and complexes with lipoproteins have been studied by various techniques—X-ray [27], EM [26, 82, 83], NMR [84], SPR [85, 86], FRET [87, 88], immuno-study [26, 82], AFM [89], and MD simulations [65, 90–92].

### CETP Crystal Structure

**Fig. 1:**
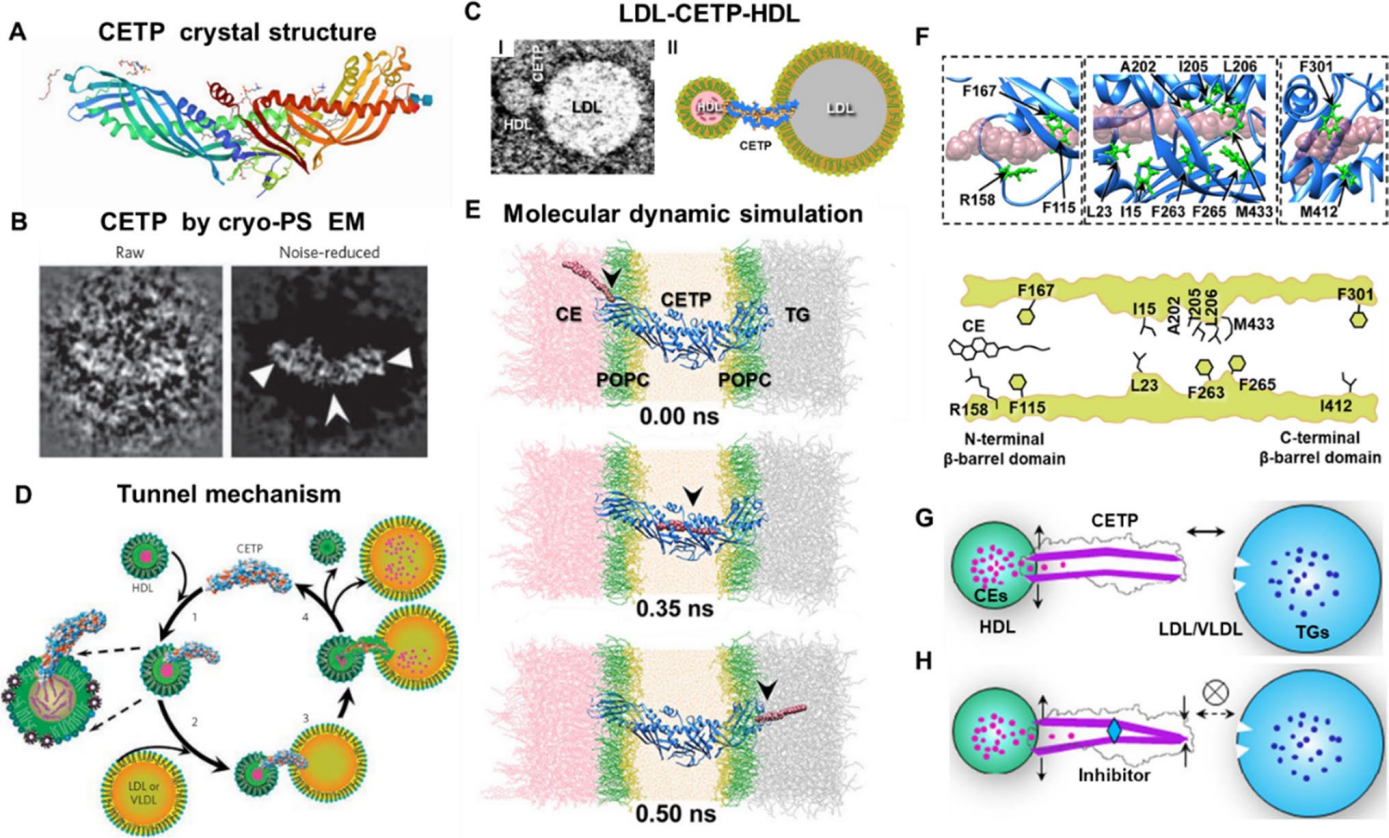
The molecular insights into the structure-based functions of cholesteryl ester transfer protein (CETP) in lipid transport. (**A**) The crystal structure of CETP (PDB: 2OBD) [27]. (**B**) A representative high-resolution cryo-positive-staining (cryo-PS) EM image (raw vs. denoised) of an individual CETP molecule (non-averaging), in which the β-sheets indicated by arrowheads. Reproduced with permission [26]. (**C**) A presentative optimized negative-staining (OpNS) EM image of an individual ternary complex of HDL-CETP-LDL (left) and its corresponding schematics (right). Reproduced with permission [90]. (**D**) Tunnel mechanism of CETP supported by the observed ternary complex [26], in which the CETP N-terminal β-barrel domain penetrates the HDL surface, which stimulates the C-terminal β-barrel domain penetrates LDL or VLDL surface in forming a ternary complex for CE transfer. Reproduced with permission [26]. (**E**) Validating the tunnel mechanism by all-atom molecular dynamics (MD) simulation. A series of snap-shot images of a CE molecule in its transferring process through the center tunnel of a CETP molecule. Reproduced with permission [90]. (**F**) MD simulations elucidated the key residues that might regulate CE transfer due to its strong interactions (top). These residues were highlighted by a cartoon. Reproduced with permission [90] (**G**) Before CETP inhibition, once the distal end of CETP interacts with HDL, the CEs were up-taken into CETP, which produces a conformational change and increases the binding affinity to LDL or VLDL. Reproduced with permission [45]. (**H**) After CETP inhibition, CEs were up-taken into one distal end which triggers a conformational change at the opposite end through the inhibitor (shown in cyan diamond), which decreased the binding affinity to other classes of lipoproteins. Reproduced with permission [45]

In the crystal structure of CETP (PDB: 2OBD) at 2.2 Å resolution [27], CETP displays an elongated-banana shape with dimensions of 135 Å × 30 Å × 35 Å (Fig. 1A) that can be divided into four domains as predicted [6, 53], i.e., a central β-sheet and a C-terminal extension that are sandwiched between two β-barrel domains. Within CETP, there is a 60-Å-long tunnel that contains two CEs, which are highly hydrophobic and a plug comprising phosphatidylcholine (PLs), which is amphiphilic. The observations that (i) CETP has a high binding affinity for HDL, (ii) a surface curvature similar to that of HDL, and (iii) two PLs plugged into the center cavity, supports the shuttle model [36]. When the CEs are transported through CEPT, the tunnel opens with concerted conformational change of the flexible helix or a mobile flap, which provides adequate space for CE transport [27].

### EM Structure

The structure of CETP has been also determined by cryo-electron microscopy (cryo-EM) [42], cryo-positive-staining (cryo-PS) [26], and negative-staining (NS) EM images [26, 42], which confirmed the crystal structure, conventional single-particle averaged 3D reconstruction [26], and IPET individual-molecule 3D reconstructions [42]. For instance, the high-resolution cryo-PS, which allowed the direct imaging of a single CETP in solution, confirmed the banana-shaped structure with a dimension of 125 Å × 30 Å. The denoised image contained features of individual molecules remarkably similar to the crystal structure (Fig. 1B), i.e., parallel fringes that are well-matched to the β-sheet strands within the N- and C-terminal β-barrel domains [26]. The averaged 3D reconstruction from thousands of CETP particles in solution showed that some loops at the distal portions of β-barrel domains appearing outside the EM density map envelope [26], confirmed that these regions are flexible. This structural plasticity was confirmed by IPET 3D reconstruction from each individual CETP particle in solution [42], which is similar to PLTP [41] and confirmed by molecular dynamics simulations [93].

### MD Simulation

For a deeper understanding of CETP structural dynamics at atomic level, all-atom MD simulations in an aqueous solution have been used [91]. These studies confirmed the distal portion flexibility [26], wherein the N-terminal β-barrel domain is significantly greater in solution than in the CETP crystal [91]. Moreover, the distal end of the C-terminal β-barrel domain expanded with the hydrophilic surface increasing more than the hydrophobic surface, in which a new surface pore was generated in this domain [91]. This surface pore and all cavities in CETP are stable, consistent with the occurrence of a continuous tunnel within CETP by connecting cavities in solution [91, 93]. Moreover, the analysis of the mechanical behavior of CETP by the molecular dynamics simulations showed, that a twisting force on the β-barrel domains and cavity formation inside the distal ends of the molecule causes these pores to connect to the hydrophobic, continuous, central cavities to form a tunnel is the conduit, similar to the Chinese finger trap model, through which HDL-CE transfer to LDL or VLDL thereby reducing HDL size [26]. The force could be generated by stimulated interaction of the C-terminal β-barrel domain with LDL or VLDL. By squeezing the molecule from two distal ends, the isolated central cavities connect and form a continuous tunnel via turning of β-barrel directions. The microsecond-long atomistic MD simulations and normal mode analysis [93] confirmed the “open state” of CETP under a twisting motion in CETP, i.e., the formation of a continuous tunnel between the two distal ends of CETP. CE transfer through CETP driven by the force undergoes significant bending–unbending during which CETP “senses” lipoproteins and structural plasticity under thermal dynamics in solution [93].

## The Binary Complex of CETP-Lipoprotein

### The Binary Complex of CETP-HDL

NS EM has been used to determine the structure and conformation of CETP incubated with HDL [26]. Contrary to the conformation in which concave surface of CETP aligns with the convex surface of HDL as predicted by crystal study [27] and MD simulations [94], the spherical HDL particles contained a surface single protruding feature in forming a lollipop-candy-shaped structure [26], an observation confirmed later [49, 82]. The protrusion indicated the CETP distal end (~48 ± 10 Å) inserted into the HDL surface or partitions tangentially into the HDL lipid surface [26, 27, 95]. Immuno-EM experiments [26] showed that CETP is oriented toward HDL via its N-terminal domain, which is highly hydrophobic as previously hypothesized [96]. A study of the linker insertion scanning mutagenesis showed that the four regions in the CETP N-terminus are essential to lipoprotein recognition [96]. Observations of CETP binding to the edge of discoidal HDL implicate APOA1 in CETP-HDL interactions [97, 98]. However, the number of CETP molecules could be much greater than the number of HDL-containing apoA-I molecules, which challenges the protein-to-protein binding hypothesis [26, 49]. Moreover, the surface curvature strongly correlates with the number of bound CETP molecules, suggesting that protein–lipid interaction mediated binding. This conclusion was further confirmed by the observation that a lollipop-candy structure is also seen in incubations of CETP with protein-free liposomes [49]. MD simulations [99] of the mechanism for CETP penetration into HDL showed that the flexible regions of CETP, i.e., flap 5 (Ω5 residues Ser90- Asp110) and flap 6 (Ω6 residues Leu150-Phe170) at the N barrel domain end of CETP, play an important anchoring role in the penetration and settling of CETP in HDL. Upon penetration, the distance between Trp106 in flap 5 and Trp162 in flap 6 was increased, suggesting the Ω5 and Ω6 are pulled apart giving rise to the CETP tunnel opening [99].

### Binary Complexes of CETP-LDL and CETP-VLDL

The structure and morphology of the CETP incubated with plasma LDL and VLDL, respectively, were investigated by NS EM [26] and cryo-electron tomography (cryo-ET) IPET 3D reconstruction [35]. The major observations were (i) a single CETP protruded from the LDL surface as a lollipop-candy shaped binary complex; (ii) No CETP molecule bridging two HDLs, two LDLs, or two VLDLs were observed; (iii) more than one CETP molecule occasionally attached to VLDL particle surface, which may be due to greater variety of VLDL apolipoproteins; (iv) The C-terminal β-barrel domain antibody, H300, blocked the CETP-LDL interaction but not CETP-HDL interaction. These observations suggest that the hydrophobic distal end of the CETP N-terminal domain preferentially interacts with HDL surface lipids, and that the distal end of the C-terminal β-barrel domain inserts into LDL and VLDL surfaces via protein–protein interaction. The structural determinants of this specificity may be their larger size and polyhedral shape, which is characterized by less curvature of surface lipid and a lower hydrophobicity which reduces the energy of binding to the hydrophobic CETP N-terminus [26]. The same mechanism underlies CETP binding to IDL [39]. These studies suggesting that CETP binding to LDL/VLDL occurs via protein–protein interaction is consistent with the observed preferential orientation of CETP during directional transfer of CE from HDL to VLDL [100].

## The Ternary Complex of CETP-Lipoproteins

In the CETP shuttle hypothesis [29], CETP undergoes diffusional collision with lipoproteins thereby forming binary complexes that mediate bidirectional lipid exchanges between the lipoproteins and CETP. As a result, the ternary complex should not have existed. Upon multiple repeats of the process [101, 102], the lipids exchange between the lipoproteins. Because of the good fit of the concave surface curvature of CETP and HDL surface curvature, the crystal structure of CETP is consistent with the shuttle mechanism and the interaction of CETP with only one lipoprotein particle at a time [27]. Moreover, CETP transfers lipids through properties of its C-terminus domain, which orders lipids through disorder-to-order transitions in its secondary structure. The CETP carboxy-terminal peptides have a stable α-helix conformation that facilitates formation of small homogeneous micelle-like structures that moves lipids through an aqueous interface within reasonable thermodynamic parameters. [83, 103].

In contrast, the EM studies of a CETP-lipoprotein complex provided evidence [26] for the ternary complex (HDL-CETP-LDL) structure (Fig. 1C), which corroborates the tunnel hypothesis (Fig. 1D) predicted nearly four decades ago [30]. The details include the images of the ternary complex showing the CETP N-terminal domain penetrating the HDL-phospholipid surface monolayer to a depth of ~17–28 Å and reaching the HDL-CE core [26], while the C-terminal domain only penetrates LDL or VLDL surface to a depth of 20–25 Å. Further study showed that the CETP binds HDL through surface-hydrophobic interactions [49], while binding to LDL/VLDL occurs via protein–protein interactions [49]. Moreover, MD simulations revealed that the N-terminal phospholipid pore, ~60 Å from the end, is adjacent to the HDL surface, which permits the possibility of surface lipid transfer [90, 99]. This pore could be important for surface phospholipid transfer when the size of HDL decreases, e.g., during CE efflux, thus potentially stabilizing the increasing or decreasing curvature of the HDL surface. EM studies from other groups did not reveal the ternary complex [82] and invoked the so-called absent of evidence to conclude that ternary complex formation is not required for CE transfer among lipoproteins [82]. However, this study is consistent with both the tunnel and shuttle mechanism and does not propose a new mechanism to explain their immuno-TEM and biophysical studies. Currently, the debate over whether CETP is a lipid tube or shuttle is unresolved. Hence, the mechanism of CETP-mediated lipid transfer remains to be further studied for better verification.

## Lipid Transfer Process

MD simulations have been used to investigate the details of lipid transfer through CETP [104]. A computer-based mechanical analysis showed that the distal portions of CETP β-barrel domains are highly flexible, permitting the hypothesized large-scale conformational excursion needed to form a continuous tunnel for CE entry and exit by connecting internal small cavities and a central cavity [91]. According to one MD simulation, a hydrophobic tunnel inside CETP is wide enough for a CE molecule to transfer through the entire molecule (Fig. 1E). Analyses of the detailed interactions revealed several residues that are critical for CETP function. This observation may provide clues that guide the design of more effective of CETP inhibitors for treatment of ASCVD [90]. MD simulation of the tunnel mechanism for TG transfer between lipoproteins by CETP [65] showed that the TGs are also transferred in the CETP core tunnel, and that the binding affinity of TGs in the CETP hydrophobic tunnel is higher than that of CEs. Thus, TG transfer through the CETP hydrophobic tunnel is slower than that of CE.

To probe the transfer pathway, a CE molecule was pulled to the surface pore at the CETP N-terminal distal end under forces ranging from 6–22 kcal/mol/Å [90]. The MD simulations, which were repeated four times under each driving force, confirmed that a CE molecule transfers through the entire CETP molecule and yielded 72 different transfer times [90]. In the simulation, several residues, such as Ile15, Leu23, Ala202, Ile205, Leu206, Phe263, Phe265, and Met433 (Fig. 1F), surrounding the narrow region (within ~10 Å range) might participate in physical contact with the CE molecule and contribute to this local high energy barrier. In addition to the above energy barrier, a low energy well was found within both the N-terminal and C-terminal β-barrel domains [90]. Phe115, Arg158, and Phe167 contributed the most energy to the N-terminal energy well (Fig. 1F), which may sequester CE from the CE pool into the CETP tunnel. Phe301 and Met412 contributed the most energy to the C-terminal energy well (Fig. 1F), which may orient or facilitate the rotation of the CE steroid rings to allow exit from the C-terminal pore. Moreover, the simulations indicated that it is possible for a hydrophobic CE molecule to transfer through an entire CETP via a continuous hydrophobic tunnel between the two distal ends of CETP (Fig. 1F). The predicted CE transfer rate was 33–125 CE molecules/s/CETP [90] which is ~30 to ~100 times faster than the measured rate of 1.14–1.54 CE molecules/s/CETP that was calculated based on the experimental radiolabeled CE against the plasma CETP concentration [105, 106]. Because the time spent for CETP travel, sensing, interacting, and penetrating both HDL and LDL was not included in the predicted transfer time, a longer time should be expected for CE transfer under physiological conditions. In addition, MD simulations coupled with free-energy calculations were employed to unravel the structure of CETP in solution. Interestingly, the phospholipids induced an elastic bent-untwisted conformation of CETP, which retains neutral lipids in its core tunnel [107].

## CETP Inhibition

Given the observation of elevated concentrations of HDL-C, a negative ASCVD risk factor, in patients with CETP deficiency [71, 108], and that in rabbits, CETP inhibition increases HDL concentrations and decreases ASCVD [109], several CETP inhibitors were developed and tested as an ASCVD therapeutic [92, 108–116].

### Torcetrapib

In ASCVD patients, the CETP inhibitor, torcetrapib, increased HDL-C concentrations 72.1% and reduced LDL-C levels by 20% [14]. Despite this effect [68, 117], there were unacceptable side effects—increased systolic blood pressure, increased aldosterone and cortisol synthesis [118], and more arterial-wall endothelin expression [119], while death and ASCVD were more frequent in the group receiving torcetrapib and atorvastatin vs. atorvastatin alone [14, 120]. Torcetrapib also produced off-target toxic effects [121], which may be related to the production of dysfunctional or proatherogenic HDL-C particles [14].

### Dalcetrapib

In patients with acute coronary syndrome, dalcetrapib increased the HDL-C levels ~31–40% vs. a 4–11% increase by the placebo [15] and reduced the risk of new-onset diabetes in patients with ASCVD [122]. However, there was no reduction of primary end-point composites of ASCVD death, nonfatal myocardial infarction, unstable angina, ischemic stroke, or cardiac arrest vs. placebo [15]. Considering no benefit vs. the placebo group [11] and side effects that included increased systolic blood pressure and episodic diarrhea [15], the study was terminated.

### Evacetrapib

Evacetrapib increased HDL-C levels +133.2% and reduced LDL-C levels −13.9%. Despite the minimal side effects and the positive effects on lipoprotein levels after three months of treatment with statin co-therapy [16], ASCVD events were not reduced and the trial was terminated [16].

*Anacetrapib* Changes in HDL-C and LDL-C levels in response to anacetrapib and statin group were respectively +104% and −41% compared to the placebo group [17]. Side effects included higher systolic and diastolic blood pressure. Anacetrapib reduced ASCVD [17, 123, 124] by reducing plasma non-HDL-C concentrations, but not those for HDL-C. This finding further challenged the idea that raising HDL-C concentrations reduces ASCVD events [72, 125, 126]. Thus, the ASCVD benefit from CETP inhibition is likely a consequence of reduced LDL-C and apoB concentrations [127].

### Obicetrapib

Monotherapy with a newer CETP inhibitor TA-8995, also known as obicetrapib, [18], reduced LDL concentrations ~45.3% and raised HDL concentrations ~179% [128]. Obicetrapib, which is well tolerated, has beneficial effects on lipids and apolipoproteins, without any serious adverse effect observed in other CETP inhibitor trials [129]. As reported, obicetrapib, as a monotherapy or concurrently with a statin to improve the concentrations of HDL-C and LDL-C is a promising drug for treating ASCVD outcomes [127, 129, 130].

Subsequent trials with off-target toxic side effects of torcetrapib [121], the relatively ineffective dalcetrapib [15], and the potent inhibitor evacetrapib [16] were stopped due to the lack of efficacy in reducing ASCVD events even though the anacetrapib reduces LDL cholesterol levels and increases HDL cholesterol levels without showing neutral or adverse effects on ASCVD outcomes [17]. Despite the varied results, clinical outcomes challenge the observational results of the inverse relationship between HDL-C levels and ASCVD events [12, 68, 131] and suggest that CETP inhibition alone is not the only mechanism that produces the cardioprotective phenotype observed in CETP-deficient individuals [13••, 132]. Alternatively, HDL contains other components such as apo-C3 and apoE, which may contribute to ASCVD in different ways [133••]. The blurred mechanism of CETP inhibition led to many questions, such as whether the elevated HDL itself was harmful [134] and whether the decreased levels of non-HDL-C particles allows HDL-C particles to transport more CE out of peripheral tissues [20••]. A new generation CETP inhibitor, such as TA-8899 [129], CKD-508 [135], and MK-8262 [136], which are still under evaluation, may change the picture. Although, CETP inhibitors were developed mainly as an ASCVD therapy, other studies have shown that they reduce diabetes and improve glycemic control [137].

*Crystal structure of CETP binding to inhibitors* In 2012, crystal structures of several CETP-inhibitors in complexes were solved by X-ray crystallography. These included the CETP-Torcetrapib complex (PDB: 4F2A) at 2.6 Å resolution [138] and CETP-Compound2 complex (PDB: 4EWS) at 3.1 Å resolution [138]. The complex structures showed that although the lipids in the C-terminal pocket of the hydrophobic tunnel remain unchanged, the buried inhibitors displaced the phospholipid from the N-terminal pocket and shifted the bound cholesteryl ester in the pocket of the long hydrophobic tunnel. The position of the inhibitors is near the narrowing neck of the hydrophobic central tunnel and likely blocks the connection between the N- and C-terminal pockets [138].

### EM Structure of CETP Binding to Inhibitors

In 2017, the EM and MD simulation were used to investigate the effects of inhibitor compounds (Torcetrapib, Dalcetrapib, and Anacetrapib) on the structure of the CETP-lipoprotein complex and CETP-mediated CE transfer [45]. These three inhibitors did not alter the structure of CETP or the conformation of CETP-lipoprotein binary complexes. However, the inhibitors increased the binding ratios of the binary complexes (CETP-HDL and CETP-LDL) and decreased the binding ratios of the ternary complexes (HDL-CETP-LDL), especially those of torcetrapib and anacetrapib [45]. These high-binding efficiencies appeared to correlate with their corresponding degrees of lipid-altering efficacies observed in large clinical trials [14, 45]. The findings of more binary complexes and fewer ternary complexes reveal an alternative mechanism of inhibition in which the binding distal end to HDL triggers a conformational change at the opposite distal end resulting in a reduced binding ratio to LDL for a reduced CE transfer rate among lipoproteins (shown in Fig. 1G and H). Moreover, the increased binding affinity between CETP and HDL by an inhibitor can reduce the concentration of free CETP in plasma, which also reduces CETP activity. However, the high binding affinity between CETP and HDL could also implicate CETP and the inhibitor HDL metabolism [45], a potential source of side effects. An ideal inhibitor design should target for disabling CETP function with decreasing the CETP-HDL binding affinity.

### MD Simulations of CETP Binding Inhibitors

In the past decade, atomistic MD simulations have been used to investigate the inhibitory mechanism by studying the interactions between the inhibitors and CETP [92, 139–141]. Aijanen et al. showed the that anacetrapib was directed toward the concave surface of CETP, and especially toward the region of the N-terminal tunnel opening [139], instead of being buried deep within the CETP pocket of the long hydrophobic tunnel revealed by the crystal structure [138]. Furthermore, Jamalan et al. showed that the anacetrapib may alter the secondary structure of CETP in the C-terminal domain and destabilize CETP-lipoprotein complex for reducing the CE transport, [140] which contradicts the EM study [45]. In the EM study, the anacetrapib increased the CETP-lipoprotein binding affinity and “locked” the CETP to lipoprotein surface, thereby reducing the free CETP in plasma and CE transportation. Chirasani et al. in 2016 proposed that the hydrophobic interactions between the CETP core tunnel residues and both torcetrapib and anacetrapib play a pivotal role and that the physical exclusion of the CETP tunnel by these small inhibitor molecules is the primary mechanism of CETP inhibition [92]. However, the roles of torcetrapib and anacetrapib in this process have not yet been explained. Yang et al. in 2018 investigated the interactions between CETP and the inhibitors (torcetrapib, anacetrapib, and evacetrapib) and showed that the inhibitors induce incremental increases in CETP rigidity and decrease the stability of the hydrophobic CETP tunnel [141]. However, the relationship between rigidity and side effects of the CETP-inhibitor complex on treating ASCVD was not explained.

## The Hypothesis of CETP Inhibitory Mechanism

Based on above reviews, we proposed a hypothetical model of CE transport and CETP inhibition as follows: CETP binds to HDL surface lipids via the hydrophobic distal end of its N-terminal β-barrel domain; smaller HDL has a higher binding affinity for CETP binding due to its higher surface curvature and hydrophobicity. This interaction causes the N-terminal β-barrel domain to compress and enlarge the internal cavities to form a single tunnel and connect to the CETP central pockets, as in a Chinese finger trapper model. When this hydrophobic tunnel reaches the HDL core, the HDL CEs are diffuse into the CETP central tunnel, thereby triggering a conformational change of the C-terminal β-barrel domain and increasing the binding affinity to apoB of LDL and VLDL. Due to a higher internal pressure within the smaller size of HDLs and a lower internal pressure within the larger size of LDL/VLDLs, the CE diffuses from HDL to LDL through the CETP central tunnel. While a CETP inhibitor, such as torcetrapib, moves from the N-terminal port, the inhibitor is trapped inside the CETP central pocket and in the CETP central tunnel by its hydrophobicity and physical size. The increased size of the central cavity by inhibitor increases the CETP rigidity and tunnel size at both β-barrel domain distal ends, which further increased the binding affinity to HDL and LDL. However, the increased binding affinity to HDL also results in more free-CETPs “parking” on the surface of HDL. The benefit of the “parking” is that it reduces the free CETP concentration in plasma, leading to fewer CEs transferred from HDL to LDL, displaying as the increased HDL-C and decreased LDL-C. However, the drawback is that the tight binding of CETP-inhibitor to HDL can involve the inhibitor in HDL metabolism, thereby inducing the CETP-inhibitor complexes to accumulate in some organs, such as skin cells.

## Conclusion

CETP mediates CE transport from HDL to LDL (or VLDL) in plasma; thus, CETP inhibition seemed an ideal strategy to increase HDL-C and decrease LDL-C as an anti-ASCVD therapy. Clinically, the CETP inhibitors, such as torcetrapib, dalcetrapib, anacetrapib, and obicetrapib increase HDL-C levels. However, the anti-ASCVD effect was no different from controls and some side effects have been reported. Thus, the value of raising HDL-C levels to reduce the risk of ASCVD events has been widely questioned. One explanation is that the plasma levels of small HDLs may be inversely correlated to ASCVD risk, while the levels of large HDLs may correlate with ASCVD risk. In recent studies, some suggested that future CETP inhibitors should have lower binding affinity to HDL or LDL. Reducing the CETP interaction to HDL/LDL may prevent the CETP-bound inhibitor from interfering with the normal HDL or LDL metabolism, which in turn, would reduce the risk of side effects. Presumably, a better understanding of CETP functions and its inhibitory mechanism will guide the design of a new generation of CETP that are effective against ASCVD.

## References

[1] Shah ASV, Stelzle D, Lee KK (2018). Global burden of atherosclerotic cardiovascular disease in people living with HIV: systematic review and meta-analysis. Circulation.

[2] Glovaci D, Fan W, Wong ND (2019). Epidemiology of diabetes mellitus and cardiovascular disease. Curr Cardiol Rep.

[3] Camejo G, Waich S, Quintero G, Berrizbeitia ML, Lalaguna F (1976). The affinity of low density lipoproteins for an arterial macromolecular complex. A study in ischemic heart disease and controls. Atherosclerosis.

[4] Gordon T, Castelli WP, Hjortland MC, Kannel WB, Dawber TR (1977). High density lipoprotein as a protective factor against coronary heart disease. Am J Med.

[5] Klerkx AH, El Harchaoui K, van der Steeg WA (2006). Cholesteryl ester transfer protein (CETP) inhibition beyond raising high-density lipoprotein cholesterol levels: pathways by which modulation of CETP activity may alter atherogenesis. Arterioscler Thromb Vasc Biol.

[6] Tall AR (1993). Plasma cholesteryl ester transfer protein. J Lipid Res.

[7] Pownall HJ, Rosales C, Gillard BK, Gotto AM (2021). High-density lipoproteins, reverse cholesterol transport and atherogenesis. Nat Rev Cardiol.

[8] Wuni R, Kuhnle GGC, Wynn-Jones AA, Vimaleswaran KS (2022). A nutrigenetic update on CETP gene-diet interactions on lipid-related outcomes. Curr Atheroscler Rep.

[9] Su X, Li G, Deng Y, Chang D (2020). Cholesteryl ester transfer protein inhibitors in precision medicine. Clin Chim Acta.

[10.••] Oestereich F, Yousefpour N, Yang E, et al. The cholesteryl ester transfer protein (CETP) raises cholesterol levels in the brain. J Lipid Res. 2022;100260. **This paper investigates the impact of cholesterol metabolism on brain function, and suggest that CETP activity affects brain health through modulating cholesterol distribution and clearance.** 10.1016/j.jlr.2022.100260 PMC9464954 35921880

[11] Lim GB (2013). Lipids. Dalcetrapib raises HDL-cholesterol level, but does not reduce cardiac risk. Nat Rev Cardiol.

[12] Taheri H, Filion KB, Windle SB, Reynier P, Eisenberg MJ (2020). Cholesteryl ester transfer protein inhibitors and cardiovascular outcomes: a systematic review and meta-analysis of randomized controlled trials. Cardiology.

[13.••] Schmidt AF, Hunt NB, Gordillo-Maranon M, et al. Cholesteryl ester transfer protein (CETP) as a drug target for cardiovascular disease. Nat Commun. 2021;12(1):5640. **This paper provided genetic evidence that CETP is an effective target for CHD prevention but with a potential on-target adverse effect on age-related macular degeneration.** 10.1038/s41467-021-25703-3 PMC8463530 34561430

[14] Barter PJ, Caulfield M, Eriksson M (2007). Effects of torcetrapib in patients at high risk for coronary events. New Engl J Med.

[15] Schwartz GG, Olsson AG, Abt M (2012). Effects of dalcetrapib in patients with a recent acute coronary syndrome. N Engl J Med.

[16] Lincoff AM, Nicholls SJ, Riesmeyer JS (2017). Evacetrapib and cardiovascular outcomes in high-risk vascular disease. N Engl J Med.

[17] Bowman L, Hopewell JC, Group HTRC (2017). Effects of anacetrapib in patients with atherosclerotic vascular disease. N Engl J Med.

[18] Larsen LE, Stoekenbroek RM, Kastelein JJP, Holleboom AG (2019). Moving targets: recent advances in lipid-lowering therapies. Arterioscler Thromb Vasc Biol.

[19] Tall AR, Yvan-Charvet L, Terasaka N, Pagler T, Wang N (2008). HDL, ABC transporters, and cholesterol efflux: implications for the treatment of atherosclerosis. Cell Metab.

[20.••] Noh S, Mai K, Shaver M, et al. Emerging cholesterol modulators for atherosclerotic cardiovascular disease. Am J Med Sci. 2022;363(5):373–387. **This paper reviewed the cholesterol targeting agents, and discussed the clinical trial efficacy as well as their implications for practical use.** 10.1016/j.amjms.2021.12.011 35081404

[21] Ready JM (2021). Toward a best-in-class inhibitor of cholesteryl ester transfer protein (CETP). J Med Chem.

[22] Inazu A. CETP deficiency and concerns in CETP inhibitor development. In: The HDL Handbook. 2017;23–35.

[23] Manzanares J, Sala F, Gutiérrez MSG, Rueda FN. Biomarkers. Compr Pharmacol. 2022;693–724.

[24.•] Tall AR, Rader DJ, Kastelein JJP. Macular degeneration and CETP inhibition. JAMA Cardiol. 2022;7(7):774–775. **This study gave a worth-considering comment that suggesting that a fully-understood CETP function and inhibitory mechanism at a molecular level is needed.** 10.1001/jamacardio.2022.1276 35648418

[25] Sirtori CR, Mombelli G (2017). CETP antagonism versus agonism in cardiovascular prevention and plaque regression. Clinical Lipidology.

[26] Zhang L, Yan F, Zhang S (2012). Structural basis of transfer between lipoproteins by cholesteryl ester transfer protein. Nat Chem Biol.

[27] Qiu X, Mistry A, Ammirati MJ (2007). Crystal structure of cholesteryl ester transfer protein reveals a long tunnel and four bound lipid molecules. Nat Struct Mol Biol.

[28] Wetterau JR, Zilversmit DB (1984). A triglyceride and cholesteryl ester transfer protein associated with liver microsomes. J Biol Chem.

[29] Barter PJ, Jones ME (1980). Kinetic studies of the transfer of esterified cholesterol between human plasma low and high density lipoproteins. J Lipid Res.

[30] Ihm J, Quinn DM, Busch SJ, Chataing B, Harmony JA (1982). Kinetics of plasma protein-catalyzed exchange of phosphatidylcholine and cholesteryl ester between plasma lipoproteins. J Lipid Res.

[31] Tall A (1995). Plasma lipid transfer proteins. Annu Rev Biochem.

[32] Shrestha S, Wu BJ, Guiney L, Barter PJ, Rye KA (2018). Cholesteryl ester transfer protein and its inhibitors. J Lipid Res.

[33] Liu J, Wu H, Huang C (2019). Optimized negative-staining protocol for lipid-protein interactions investigated by electron microscopy. Methods Mol Biol.

[34.••] Zhang L, Ren G. IPET and FETR: experimental approach for studying molecular structure dynamics by cryo-electron tomography of a single-molecule structure. PLoS One. 2012;7(1):e30249. **This study provided images of the CETP interaction to lipoproteins at molecular levels.** 10.1371/journal.pone.0030249 PMC3265479 22291925

[35] Zhai X, Lei D, Zhang M (2020). LoTToR: An algorithm for missing-wedge correction of the low-tilt tomographic 3D reconstruction of a single-molecule structure. Sci Rep.

[36] de Grooth GJ, Klerkx AH, Stroes ES, Stalenhoef AF, Kastelein JJ, Kuivenhoven JA (2004). A review of CETP and its relation to atherosclerosis. J Lipid Res.

[37] Zlotnick A (2004). Viruses and the physics of soft condensed matter. Proc Natl Acad Sci U S A.

[38] Wang S-T, Minevich B, Liu J (2021). Protein lattices: structurally designed, dimensionally controlled and biologically active. Nat Commun.

[39] Lei D, Yu Y, Kuang YL, Liu J, Krauss RM, Ren G (2019). Single-molecule 3D imaging of human plasma intermediate-density lipoproteins reveals a polyhedral structure. Biochim Biophys Acta Mol Cell Biol Lipids.

[40] Lei D, Liu J, Liu H (2019). Single-molecule 3D images of "hole-hole" IgG1 homodimers by individual-particle electron tomography. Sci Rep.

[41] Zhang M, Zhai X, Li J, Albers JJ, Vuletic S, Ren G (2018). Structural basis of the lipid transfer mechanism of phospholipid transfer protein (PLTP). Biochim Biophys Acta Mol Cell Biol Lipids.

[42] Wu H, Zhai X, Lei D (2018). An algorithm for enhancing the image contrast of electron tomography. Sci Rep.

[43] Lei D, Marras AE, Liu J (2018). Three-dimensional structural dynamics of DNA origami Bennett linkages using individual-particle electron tomography. Nat Commun.

[44] Jay JW, Bray B, Qi Y (2018). IgG antibody 3D structures and dynamics. Antibodies (Basel)..

[45] Zhang M, Lei D, Peng B (2017). Assessing the mechanisms of cholesteryl ester transfer protein inhibitors. Biochim Biophys Acta Mol Cell Biol Lipids.

[46] Zhang L, Lei D, Smith JM (2016). Three-dimensional structural dynamics and fluctuations of DNA-nanogold conjugates by individual-particle electron tomography. Nat Commun.

[47] Yu Y, Kuang YL, Lei D (2016). Polyhedral 3D structure of human plasma very low density lipoproteins by individual particle cryo-electron tomography1. J Lipid Res.

[48] Zhang X, Zhang L, Tong H (2015). 3D Structural fluctuation of IgG1 antibody revealed by individual particle electron tomography. Sci Rep.

[49] Zhang M, Charles R, Tong H (2015). HDL surface lipids mediate CETP binding as revealed by electron microscopy and molecular dynamics simulation. Sci Rep.

[50] Getz GS (2018). Lipid transfer proteins: introduction to the thematic review series. J Lipid Res.

[51] Huuskonen J, Olkkonen VM, Jauhiainen M, Ehnholm C (2001). The impact of phospholipid transfer protein (PLTP) on HDL metabolism. Atherosclerosis.

[52] Schumann RR, Leong SR, Flaggs GW (1990). Structure and function of lipopolysaccharide binding protein. Science.

[53] Bruce C, Beamer LJ, Tall AR (1998). The implications of the structure of the bactericidal/permeability-increasing protein on the lipid-transfer function of the cholesteryl ester transfer protein. Curr Opin Struct Biol.

[54] Jiang XC (2018). Phospholipid transfer protein: its impact on lipoprotein homeostasis and atherosclerosis. J Lipid Res.

[55] Rao RP, Yuan C, Allegood JC (2007). Ceramide transfer protein function is essential for normal oxidative stress response and lifespan. Proc Natl Acad Sci U S A.

[56] Kanno K, Wu MK, Scapa EF, Roderick SL, Cohen DE (2007). Structure and function of phosphatidylcholine transfer protein (PC-TP)/StarD2. Biochim Biophys Acta.

[57] Ghosh R, Bankaitis VA (2011). Phosphatidylinositol transfer proteins: negotiating the regulatory interface between lipid metabolism and lipid signaling in diverse cellular processes. BioFactors.

[58] Naylor HM, Newcomer ME (1999). The structure of human retinol-binding protein (RBP) with its carrier protein transthyretin reveals an interaction with the carboxy terminus of RBP. Biochemistry.

[59] Min KC, Kovall RA, Hendrickson WA (2003). Crystal structure of human alpha-tocopherol transfer protein bound to its ligand: implications for ataxia with vitamin E deficiency. Proc Natl Acad Sci U S A.

[60] Tu A-Y, Chen H, Johnson KA, Paigen B, Albers JJ (1997). Characterization of the mouse gene encoding phospholipid transfer protein. Gene.

[61] Beamer LJ, Carroll SF, Eisenberg D (1997). Crystal structure of human BPI and two bound phospholipids at 2.4 angstrom resolution. Science.

[62] Masson D, Jiang XC, Lagrost L, Tall AR (2009). The role of plasma lipid transfer proteins in lipoprotein metabolism and atherogenesis. J Lipid Res.

[63] Drayna D, Jarnagin AS, McLean J (1987). Cloning and sequencing of human cholesteryl ester transfer protein cDNA. Nature.

[64] Luo Y, Tall AR (2000). Sterol upregulation of human CETP expression in vitro and in transgenic mice by an LXR element. J Clin Invest.

[65] Chirasani VR, Senapati S (2017). How cholesteryl ester transfer protein can also be a potential triglyceride transporter. Sci Rep.

[66] Wang X, Driscoll DM, Morton RE (1999). Molecular cloning and expression of lipid transfer inhibitor protein reveals its identity with apolipoprotein F. J Biol Chem.

[67] Gautier T, Masson D, de Barros JP (2000). Human apolipoprotein C-I accounts for the ability of plasma high density lipoproteins to inhibit the cholesteryl ester transfer protein activity. J Biol Chem.

[68] Tall AR, Rader DJ (2018). Trials and tribulations of CETP inhibitors. Circ Res.

[69] Armitage J, Holmes MV, Preiss D (2019). Cholesteryl ester transfer protein inhibition for preventing cardiovascular events: JACC review topic of the week. J Am Coll Cardiol.

[70] Matsuura F, Wang N, Chen WG, Jiang XC, Tall AR (2006). HDL from CETP-deficient subjects shows enhanced ability to promote cholesterol efflux from macrophages in an apoE- and ABCG1-dependent pathway. J Clin Invest.

[71] Brown ML, Inazu A, Hesler CB (1989). Molecular basis of lipid transfer protein deficiency in a family with increased high-density lipoproteins. Nature.

[72] Inazu A, Brown ML, Hesler CB (1990). Increased high-density lipoprotein levels caused by a common cholesteryl-ester transfer protein gene mutation. N Engl J Med.

[73] Curb JD, Abbott RD, Rodriguez BL (2004). A prospective study of HDL-C and cholesteryl ester transfer protein gene mutations and the risk of coronary heart disease in the elderly. J Lipid Res.

[74] Zhong S, Sharp DS, Grove JS (1996). Increased coronary heart disease in Japanese-American men with mutation in the cholesteryl ester transfer protein gene despite increased HDL levels. J Clin Invest.

[75] Thompson A, Di Angelantonio E, Sarwar N (2008). Association of cholesteryl ester transfer protein genotypes with CETP mass and activity, lipid levels, and coronary risk. JAMA.

[76] Rosenson RS, Brewer HB, Ansell BJ (2016). Dysfunctional HDL and atherosclerotic cardiovascular disease. Nat Rev Cardiol.

[77] Rao R, Albers JJ, Wolfbauer G, Pownall HJ (1997). Molecular and macromolecular specificity of human plasma phospholipid transfer protein. Biochemistry.

[78] Tall AR, Krumholz S, Olivecrona T, Deckelbaum RJ (1985). Plasma phospholipid transfer protein enhances transfer and exchange of phospholipids between very low density lipoproteins and high density lipoproteins during lipolysis. J Lipid Res.

[79] Jauhiainen M, Metso J, Pahlman R, Blomqvist S, van Tol A, Ehnholm C (1993). Human plasma phospholipid transfer protein causes high density lipoprotein conversion. J Biol Chem.

[80] Asztalos BF (2004). Study HDLAT: High-density lipoprotein metabolism and progression of atherosclerosis: new insights from the HDL Atherosclerosis Treatment Study. Curr Opin Cardiol.

[81] Oram JF, Wolfbauer G, Tang C, Davidson WS, Albers JJ (2008). An amphipathic helical region of the N-terminal barrel of phospholipid transfer protein is critical for ABCA1-dependent cholesterol efflux. J Biol Chem.

[82] Lauer ME, Graff-Meyer A, Rufer AC (2016). Cholesteryl ester transfer between lipoproteins does not require a ternary tunnel complex with CETP. J Struct Biol.

[83] Garcia-Gonzalez V, Gutierrez-Quintanar N, Mendoza-Espinosa P, Brocos P, Pineiro A, Mas-Oliva J (2014). Key structural arrangements at the C-terminus domain of CETP suggest a potential mechanism for lipid-transfer activity. J Struct Biol.

[84] Korhonen A, Ala-korpela M, Liinamaa MJ, Jokisaari J, Kesäniemi YA, Savolainen MJ (1997). Assessment of cholesteryl ester transfer protein function in lipoprotein mixtures by1H NMR spectroscopy. NMR Biomed.

[85] Rajaram OV, Sawyer WH (1996). Characterisation of lipid-protein interactions using a surface plasmon resonance biosensor. Biochem Mol Biol Int.

[86] Deguchi H, Banerjee Y, Elias DJ, Griffin JH (2016). Elevated CETP lipid transfer activity is associated with the risk of venous thromboembolism. J Atheroscler Thromb.

[87] Ferretti G, Bacchetti T, Negre-Salvayre A, Salvayre R, Dousset N, Curatola G (2006). Structural modifications of HDL and functional consequences. Atherosclerosis.

[88] Ginsburg BE, Zetterstrom R (1977). High density lipoprotein concentrations in newborn infants. Acta Paediatr Scand.

[89] Lemkadem B, Saulnier P, Boury F, Proust JE (1999). Interfacial behavior of HDL3 spread at air/water interface. II. Structural analysis by AFM. Colloids Surf B: Biointerfaces.

[90] Lei D, Rames M, Zhang X, Zhang L, Zhang S, Ren G (2016). Insights into the tunnel mechanism of cholesteryl ester transfer protein through all-atom molecular dynamics simulations. J Biol Chem.

[91] Lei D, Zhang X, Jiang S (2013). Structural features of cholesteryl ester transfer protein: a molecular dynamics simulation study. Proteins.

[92] Chirasani VR, Sankar R, Senapati S (2016). Mechanism of inhibition of cholesteryl ester transfer protein by small molecule inhibitors. J Phys Chem B.

[93] Chirasani VR, Revanasiddappa PD, Senapati S (2016). Structural plasticity of cholesteryl ester transfer protein assists the lipid transfer activity. J Biol Chem.

[94] Koivuniemi A, Vuorela T, Kovanen PT, Vattulainen I, Hyvonen MT (2012). Lipid exchange mechanism of the cholesteryl ester transfer protein clarified by atomistic and coarse-grained simulations. PLoS Comput Biol.

[95] Hall J, Qiu X (2011). Structural and biophysical insight into cholesteryl ester-transfer protein. Biochem Soc Trans.

[96] Wang S, Deng LP, Brown ML, Agellon LB, Tall AR (1991). Structure-function studies of human cholesteryl ester transfer protein by linker insertion scanning mutagenesis. Biochemistry.

[97] Bruce C, Davidson WS, Kussie P (1995). Molecular determinants of plasma cholesteryl ester transfer protein binding to high density lipoproteins. J Biol Chem.

[98] Dergunov AD, Shabrova EV, Dobretsov GE (2014). Cholesteryl ester diffusion, location and self-association constraints determine CETP activity with discoidal HDL: excimer probe study. Arch Biochem Biophys.

[99] Cilpa-Karhu G, Jauhiainen M, Riekkola ML (2015). Atomistic MD simulation reveals the mechanism by which CETP penetrates into HDL enabling lipid transfer from HDL to CETP. J Lipid Res.

[100] Guerin M, Egger P, Soudant C (2002). Cholesteryl ester flux from HDL to VLDL-1 is preferentially enhanced in type IIB hyperlipidemia in the postprandial state. J Lipid Res.

[101] Barter PJ, Hopkins GJ, Calvert GD (1982). Transfers and exchanges of esterified cholesterol between plasma lipoproteins. Biochem J.

[102] Hesler CB, Tall AR, Swenson TL, Weech PK, Marcel YL, Milne RW (1988). Monoclonal antibodies to the Mr 74,000 cholesteryl ester transfer protein neutralize all of the cholesteryl ester and triglyceride transfer activities in human plasma. J Biol Chem.

[103] Garcia-Gonzalez V, Mas-Oliva J (2011). Amyloidogenic properties of a D/N mutated 12 amino acid fragment of the C-terminal domain of the Cholesteryl-Ester Transfer Protein (CETP). Int J Mol Sci.

[104] Deng S, Liu J, Niu C (2022). HDL and cholesterol ester transfer protein (CETP). Adv Exp Med Biol.

[105] Hannuksela M, Marcel YL, Kesäniemi YA, Savolainen MJ (1992). Reduction in the concentration and activity of plasma cholesteryl ester transfer protein by alcohol. J Lipid Res.

[106] Lassel TS, Guerin M, Auboiron S, Chapman MJ, Guy-Grand B (1998). Preferential cholesteryl ester acceptors among triglyceride-rich lipoproteins during alimentary lipemia in normolipidemic subjects. Arterioscler Thromb Vasc Biol.

[107] Revanasiddappa PD, Sankar R, Senapati S (2018). Role of the bound phospholipids in the structural stability of cholesteryl ester transfer protein. J Phys Chem B.

[108] Prinz WA (2014). Bridging the gap: membrane contact sites in signaling, metabolism, and organelle dynamics. J Cell Biol.

[109] Barter PJ, Brewer HB, Chapman MJ, Hennekens CH, Rader DJ, Tall AR (2003). Cholesteryl ester transfer protein: a novel target for raising HDL and inhibiting atherosclerosis. Arterioscler Thromb Vasc Biol.

[110] Mabuchi H, Nohara A, Inazu A (2014). Cholesteryl ester transfer protein (CETP) deficiency and CETP inhibitors. Mol Cells.

[111] Marsche G, Saemann MD, Heinemann A, Holzer M (2013). Inflammation alters HDL composition and function: implications for HDL-raising therapies. Pharmacol Ther.

[112] Rashid S (2020). Lower LDL is better – can this be achieved with CETP inhibition therapy?. Expert Rev Cardiovasc Ther.

[113] Barter PJ, Rye KA (2012). Cholesteryl ester transfer protein inhibition as a strategy to reduce cardiovascular risk. J Lipid Res.

[114] Morehouse LA, Sugarman ED, Bourassa PA (2007). Inhibition of CETP activity by torcetrapib reduces susceptibility to diet-induced atherosclerosis in New Zealand White rabbits. J Lipid Res.

[115] Rennings AJ, Stalenhoef A (2008). JTT-705: is there still future for a CETP inhibitor after torcetrapib?. Expert Opin Investig Drugs.

[116] Xie L, Li J, Xie L, Bourne PE (2009). Drug discovery using chemical systems biology: identification of the protein-ligand binding network to explain the side effects of CETP inhibitors. PLoS Comput Biol.

[117] Brousseau ME, Schaefer EJ, Wolfe ML (2004). Effects of an inhibitor of cholesteryl ester transfer protein on HDL cholesterol. N Engl J Med.

[118] Forrest MJ, Bloomfield D, Briscoe RJ (2008). Torcetrapib-induced blood pressure elevation is independent of CETP inhibition and is accompanied by increased circulating levels of aldosterone. Br J Pharmacol.

[119] Simic B, Hermann M, Shaw SG (2012). Torcetrapib impairs endothelial function in hypertension. Eur Heart J.

[120] Keene D, Price C, Shun-Shin MJ, Francis DP (2014). Effect on cardiovascular risk of high density lipoprotein targeted drug treatments niacin, fibrates, and CETP inhibitors: meta-analysis of randomised controlled trials including 117,411 patients. BMJ.

[121] Vergeer M, Bots ML, van Leuven SI (2008). Cholesteryl ester transfer protein inhibitor torcetrapib and off-target toxicity: a pooled analysis of the rating atherosclerotic disease change by imaging with a new CETP inhibitor (RADIANCE) trials. Circulation.

[122] Schwartz GG, Leiter LA, Ballantyne CM (2020). Dalcetrapib reduces risk of new-onset diabetes in patients with coronary heart disease. Diabetes Care.

[123] Bowman L, Hopewell JC, Chen F, Wallendszus K, Stevens W (2018). Effects of anacetrapib in patients with atherosclerotic vascular disease. J Vasc Surg.

[124] Cannon CP, Shah S, Dansky HM (2010). Safety of anacetrapib in patients with or at high risk for coronary heart disease. N Engl J Med.

[125] Assmann G, Schulte H (1992). Relation of high-density lipoprotein cholesterol and triglycerides to incidence of atherosclerotic coronary artery disease (the PROCAM experience). Prospective Cardiovascular Munster study. Am J Cardiol.

[126] Ferri N, Corsini A, Sirtori CR, Ruscica M (2018). Present therapeutic role of cholesteryl ester transfer protein inhibitors. Pharmacol Res.

[127] Nurmohamed NS, Ditmarsch M, Kastelein JJP. CETP-inhibitors: from HDL-C to LDL-C lowering agents? Cardiovasc Res 2021;cvab350. 10.1093/cvr/cvab350 PMC9648826 34849601

[128] Kastelein JJ, Duivenvoorden R, Deanfield J (2011). Rationale and design of dal-VESSEL: a study to assess the safety and efficacy of dalcetrapib on endothelial function using brachial artery flow-mediated vasodilatation. Curr Med Res Opin.

[129] Hovingh GK, Kastelein JJ, van Deventer SJ (2015). Cholesterol ester transfer protein inhibition by TA-8995 in patients with mild dyslipidaemia (TULIP): a randomised, double-blind, placebo-controlled phase 2 trial. Lancet.

[130] Banerjee S, De A (2021). Pathophysiology and inhibition of cholesteryl ester transfer protein for prevention of cardiovascular diseases: An update. Drug Discov Today.

[131] Krishna R, Gheyas F, Liu Y (2017). Chronic administration of anacetrapib is associated with accumulation in adipose and slow elimination. Clin Pharmacol Ther.

[132] van Capelleveen JC, van der Valk FM, Stroes ES (2016). Current therapies for lowering lipoprotein (a). J Lipid Res.

[133.••] Furtado JD, Ruotolo G, Nicholls SJ, Dullea R, Carvajal-Gonzalez S, Sacks FM. Pharmacological inhibition of CETP (cholesteryl ester transfer protein) increases HDL (high-density lipoprotein) that contains ApoC3 and other HDL subspecies associated with higher risk of coronary heart disease. Arterioscler Thromb Vasc Biol. 2022;42(2):227–237. **This study suggested the increased plasma HDL by CETP inhibition increased dysfunctional subspecies such as HDL that contains both apoC3 and apoE, a complex associated with higher coronary heart disease risk.** 10.1161/ATVBAHA.121.317181 PMC8785774 34937388

[134] Zanoni P, Khetarpal SA, Larach DB (2016). Rare variant in scavenger receptor BI raises HDL cholesterol and increases risk of coronary heart disease. Science.

[135] Lee JM, Lee YJ, Kwon NY, Ryu KH. Old target, but new drug: 2nd generation cetp inhibitor, CKD-508. Atherosclerosis. 2020;315.

[136] Vachal P, Duffy JL, Campeau LC (2021). Invention of MK-8262, a cholesteryl ester transfer protein (CETP) inhibitor backup to anacetrapib with best-in-class properties. J Med Chem.

[137] Dangas K, Navar AM, Kastelein JJP (2022). The effect of CETP inhibitors on new-onset diabetes: a systematic review and meta-analysis. Eur Heart J Cardiovasc Pharmacother.

[138] Liu S, Mistry A, Reynolds JM (2012). Crystal structures of cholesteryl ester transfer protein in complex with inhibitors. J Biol Chem.

[139] Aijanen T, Koivuniemi A, Javanainen M, Rissanen S, Rog T, Vattulainen I (2014). How anacetrapib inhibits the activity of the cholesteryl ester transfer protein? Perspective through atomistic simulations. PLoS Comput Biol.

[140] Jamalan M, Zeinali M, Ghaffari MA (2016). A molecular dynamics investigation on the inhibition mechanism of cholesteryl ester transfer protein by Anacetrapib. Med Chem Res.

[141] Yang Z, Cao Y, Hao D, Yuan X, Zhang L, Zhang S (2018). Binding profiles of cholesterol ester transfer protein with current inhibitors: a look at mechanism and drawback. J Biomol Struct Dyn.

